# Ziemssen’s Cyclopaedia, Vol. III

**Published:** 1875-08

**Authors:** 


### Books Reviewed

*Cyclopædia of the Practice of Medicine.* Edited by Dr. H. von Ziemssen. Vol. III. Chronic Infectious Diseases. American Translation Edited by Albert H. Buck, M. D. New York: Wm. Wood & Co., 1875.

The topics discussed in this volume are under three heads: Syphilis by Prof. Baumler, Infection by Animal Poison by Prof. Bollinger, and Diseases from Migratory Parasites by Prof. Heller.

Syphilis occupies nearly one-half of the work, and is an able resumé of the more recent and best views upon the subject. The author does not give credence to the stories of its American origin but places its birth place in Southern Europe in the latter part of the fifteenth century.

The history and geographical distribution of syphilis are considered somewhat in detail, occupying some twenty pages. The general features of the disease are summed up in a brief but concise manner, occupying five pages in their consideration. This is followed by a consideration of the various stages. The distinguishing features of the primary, secondary and tertiary stages are given as set forth by Ricord. The objections to these arbitrary divisions are that an investigation of the pathological anatomy of the disease reveals the fact that the classification of Ricord is neither correct anatomically nor chronologically. It has been shown by Virchow that the mild symptoms of hyperaemia and exudations, or the more severe one of destructive ulceration and tubercular deposits may occur at any stage of the disease, or may in fact accompany each other. Believing, however, that the distinctions of Ricord in a modified form presents many attractive features in a clinical point of view, the author, after a consideration of the pathological characteristics and therapeutical indications of the disease in its various stages, presents a classification of a similar nature. In this arrangement a fourth stage is presented that of *confirmed syphilitic marasmus.* The modifications and variety of form in these various stages the author says may be due to a difference in the original virus or the individual characteristics. To this latter view we are inclined to attach the most importance; the former is at least a matter of conjecture, while all practitioners have noticed that the individual constitution of the patient will modify the action of the poison. The various modes of conveying the poison, by kissing, by means of drinking cup, pipes, etc., in obstetrical cases, and in surgical operations, and by vaccination are well treated. In the matter of vaccination the weight of opinion seems to be that the virus is conveyed by the blood and not by the vaccine lymph, still it seems eminently proper to exercise great choice in the selection of vaccine virus. An interesting discussion occupying some twenty-three pages is upon the doctrines of Unity and Duality.

In his conclusion the author says with much truth, "In any given case the inquiry should be: *Was there any syphilis in the infecting source?* The question of Unity and Duality have turned too much upon the characteristic appearance of the sore, and too little attention has been paid by many who are ever ready to discuss this point to the pathological conditions obtained under the two circumstances." We heartily agree with the author when he says that the theory of chancre and syphilis may be formulated under the following statement: "The chancre (soft chancre) is a local contagious affection which is inoculable upon the bearer, and upon others both healthy and syphilitic, to an almost unlimited extent. It is developed without incubation within twenty-four hours. Through resorption an irritation of the adjacent glands takes place, which has an acute inflammatory character, and usually leads to suppuration, *but is not followed by constitutional syphilis.* The description of the true syphilitic chancre which follows is full and true to nature, and the exceptional diveations which occur, and which have given rise to many disputes are well noted. The microscopic appearance of primary affections, the different processes in the various stages in the evolution of the disease, and the affections of the various parts are well described. Sections upon the diagnosis, prognosis and treatment of the disease close this portion of the work. They are in entire harmony with the balance of the section, that upon treatment we have noticed in an other portion of this JOURNAL, (page 21) and forbear dwelling further upon the subject.

Infection by animal poisons is an interesting section by Prof. Bollinger, who has given especial attention to the diseases of animals.

The various diseases, glanders, anthrax, hydrophobia and the foot-and mouth disease are considered first in the animal and then in man. A chapter upon infection from the bite or sting of poisonous insects or animals closes Prof. Bollinger's portion of the work. Though not so much practical interest as the preceding portion it is nevertheless full of instructive matter and forms a valuable portion of the work.

Diseases from migratory parasites receive attention from Prof. Heller. Under this head are considered echinococcus, cystericus cellulosæ and trichinæ. The latter of these subjects seems to be of the most general interest, and it as well as the other two subjects are well considered. The illustrations are well selected, and serve admirably to elucidate the text. As a whole, volume three serves well to maintain the expectations which the former two volumes called forth.

